# Fabrication of a Carboxylate Cellulose Nanocrysal-Silica-TiO_2_ Aerogel for Enhanced Photocatalytic Degradation of Methylene Blue

**DOI:** 10.3390/ma18204702

**Published:** 2025-10-14

**Authors:** Nduduzo Lungisani Khumalo, Samson Masulubanye Mohomane, Vetrimurugan Elumalai, Tshwafo Elias Motaung

**Affiliations:** 1 Department of Chemistry, University of Zululand, KwaDlangezwa Campus, KwaDlangezwa 3886, South Africa; 2 Department of Hydrology, University of Zululand, KwaDlangezwa Campus, KwaDlangezwa 3886, South Africa; 3 Department of Chemistry and Chemical Technology, Sefako Makgatho Health Science University, P.O. Box 94, Medunsa 0204, South Africa

**Keywords:** sugarcane bagasse, cellulose, photocatalytic degradation, adsorption

## Abstract

The insistent presence of detrimental chemical dyes, such as methylene blue (MB), in aquatic ecosystems creates a significant environmental fear that requires the development of innovative and effective remediation methods. This study examines the production and application of a novel carboxylate cellulose nanocrystal-silica-titanium dioxide (CCNC-silica-TiO_2_) hybrid composite aerogel designed to enhance the photocatalytic degradation of methylene blue (MB). Carboxylic groups were incorporated into cellulose nanocrystals (CNCs) derived from sugarcane bagasse (SCB) waste to improve their dye adsorption capacity. The CCNCs were later incorporated into a silica aerogel matrix using a sol–gel method, followed by the introduction of TiO_2_ nanoparticles. Characterization techniques, including FTIR and XRD, confirmed the successful chemical functionalization and composite synthesis. SEM analysis revealed a highly porous three-dimensional architecture, whilst BET surface area assessment showed that the CCNC-SiO_2_-TiO_2_ aerogel possessed a significant specific surface area of 448.69 m^2^/g. Under ultraviolet light, the hybrid aerogel demonstrated remarkable photocatalytic performance, achieving a 93% degradation rate of methylene blue, far above the 22% recorded in a CCNC-silica control. The degradation kinetics followed a pseudo-first-order model. The composite demonstrated significant reusability, maintaining over 70% efficiency after five consecutive cycles. The findings indicate that the adsorptive capacity of carboxylate CNCs, together with the photocatalytic efficiency of TiO_2_, improves the efficacy, stability, and longevity of the CCNC-SiO_2_-TiO_2_ aerogel in wastewater treatment.

## 1. Introduction

The environmental problem of water pollution from organic dyes, including methylene blue (MB), exists because these substances remain toxic while being persistent and causing severe health and ecological damage [1,2]. Methylene blue functions as a widely used dye in textile production, paper manufacturing, and leather processing. In this study, methylene blue (MB) was selected as a model organic pollutant for several key reasons. Firstly, it is representative of the thiazine class of dyes, which are extensively used and commonly found in industrial wastewater. Secondly, its high visibility and stability allow for straightforward and precise monitoring of the degradation process using UV-Vis spectroscopy. Furthermore, as a cationic dye, it serves as an ideal probe to evaluate the electrostatic interaction with the anionic carboxylate groups (-COO^−^) on the functionalized cellulose nanocrystals (CCNCs) within our composite. Its well-documented degradation pathway also makes it a standard benchmark compound for evaluating the efficacy of new photocatalytic materials [3,4]. The decomposition process of this substance creates multiple health risks, including skin irritation and DNA alterations, and severe aquatic ecosystem pollution, unless appropriate treatment occurs before release [3,4]. The increasing need for efficient wastewater treatment methods to remove these contaminants has become essential.

The removal of dyes from wastewater through adsorption, chemical oxidation, and biological treatment methods proves ineffective for treating high-concentration dye effluents [5,6]. Photocatalysis has emerged as a promising technique for converting organic contaminants into innocuous end products, utilizing sunshine or artificial illumination [7,8]. Titanium dioxide (TiO_2_) is acknowledged as an efficient photocatalyst, characterized by its stability, non-toxicity, and capability to oxidize diverse compounds when subjected to UV radiation [9]. However, its practical application is constrained by insufficient retention of dye molecules and the rapid recombination of electron-hole pairs [10].

In this context, cellulose nanocrystals (CNCs) derived from sustainable biomass offer a compelling, green alternative as a substrate for TiO_2_. CNCs exhibit superior mechanical properties, a high surface area, biocompatibility, and, most importantly, a hydroxyl-rich surface that can be readily functionalized [11]. The specific incorporation of carboxyl groups into CNCs (forming CCNCs) creates anionic sites that enhance the electrostatic adhesion of cationic dye molecules like methylene blue, effectively pre-concentrating the pollutant at the photocatalytic sites [12]. Furthermore, the integration of a silica matrix within the composite serves a critical dual purpose: it ensures the structural stability of the hybrid material and inhibits the agglomeration of TiO_2_ nanoparticles, thereby maintaining a high density of active sites [13].

This study introduces a novel ternary CCNC-SiO_2_-TiO_2_ aerogel designed to exceed the limitations of individual components and conventional composites through a unique multi-functional synergy. The distinctive characteristic of this system is that each component has a specific function that integrates effectively with the others within the 3D aerogel architecture: The carboxylate cellulose nanocrystals (CCNCs) have dual purposes beyond being a sustainable substrate. They provide a high density of anionic surface charges that serve as molecular docking sites for cationic dye pollutants such as MB, thereby pre-concentrating them at the photocatalytic sites, a function that is less pronounced in non-functionalized carbons. The silica aerogel matrix not only prevents the aggregation of TiO_2_ particles but also constructs a robust, highly porous, and thermally stable three-dimensional scaffold with an extensive surface area, facilitating the accessibility and interaction between CCNCs and TiO_2_ nanoparticles. The TiO_2_ nanoparticles, which are effectively disseminated and bound by this matrix, mineralize the colours that have been previously adsorbed. This aerogel, conversely, is a singular entity that is facile to manipulate and lacks the recovery issues associated with nanopowders. This research’s innovation resides in the integration of these materials, creating a synergistic system where CCNC promotes targeted adsorption, SiO_2_ provides an optimized porous structure, and TiO_2_ facilitates localized photocatalysis, all combined into a reusable macroscopic solid for efficient wastewater treatment [14,15].

This research investigates the development of a carboxylate cellulose nanocrystal silica titanium dioxide (CCNC-SiO_2_-TiO_2_) hybrid composite aerogel and assesses its effectiveness in the photocatalytic degradation of methylene blue dye in contaminated water. The synthesis entails the co-assembly of CCNCs, silica, and TiO_2_ nanoparticles, succeeded by vacuum drying to yield a porous aerogel structure. The composite was analyzed using scanning electron microscopy (SEM), X-ray diffraction (XRD), and Fourier transform infrared spectroscopy (FTIR). This ensured that each component was successfully integrated and enhanced our understanding of the aerogel’s structure and function.

This study evaluates the effectiveness of the CCNC-SiO_2_-TiO_2_ hybrid composite aerogel as a photocatalyst by analyzing its ability to decompose methylene blue under UV light exposure. This study examines the influence of operational parameters, such as initial dye concentration, pH, and catalyst dosage, on photocatalytic efficiency. We will evaluate the longevity and reusability of the composite aerogel throughout multiple photocatalytic cycles. This research seeks to improve hybrid materials for environmental cleanup. This study presents materials that effectively and durably eliminates organic dyes from wastewater through photocatalysis. The CCNC-SiO_2_-TiO_2_ hybrid composite aerogel aims to enhance photocatalytic efficiency, stability, and reusability by integrating the optimal characteristics of CNCs, silica, and TiO_2_. This fulfils the fundamental requirement for cleaner and safer water sources.

## 2. Materials and Methods

### 2.1. Materials

Sugarcane bagasse (SCB) waste was supplied by the sugar company in Empangeni (Felixton), KwaZulu-Natal, South Africa. Hydrochloric acid (32%) and sodium hydroxide pellets were purchased from Laboratory Supplies and used as received. Acetic acid, Sodium chlorite (80%), Ammonium persulfate, Ammonia (25%), Tertiary butyl alcohol, Titanium tetra-isopropoxide 98%, Absolute ethanol, and tetraethyl orthosilicate (98%) were obtained from Prestige Laboratories, South Africa.

### 2.2. Extraction of Cellulose

The sugarcane bagasse (SCB) was mechanically ground using a Fritsch cutting mill pulveriser 15 (FRITSCH GmbH, Bad Ems, Germany). It was then boiled in distilled water for 2 h and dried in an oven at 55 °C overnight. The dried SCB was chemically treated with 4 wt% sodium hydroxide (NaOH) at 80 °C for 1 h, followed by rinsing with distilled water, with this process repeated twice. The alkali-treated SCB was subsequently dried in an oven at 55 °C.

Next, the alkali-treated SCB underwent a bleaching process using a buffer solution consisting of 54 g of NaOH, 150 mL of acetic acid (CH_3_COOH), and 2 L of 1.7 wt% sodium chlorite (NaClO_2_) solution at 80 °C for 1 h, with this step also repeated twice. Finally, the bleached cellulose was filtered, washed with distilled water until a neutral pH was achieved, and dried overnight at 55 °C.

### 2.3. Preparation of Carboxylate Cellulose Nanocrystals

An 18 g dry mass of cellulose was dispersed into a 600 mL solution of 2 M ammonium persulfate (APS). The mixture was then ultrasonicated at a high frequency with continuous stirring for four hours at 70 °C. Following this treatment, the resulting dispersion was centrifuged at 6000 rpm with distilled water, repeating the process four times. Subsequently, the suspension was dialyzed against distilled water until the carboxylate cellulose nanocrystal (CCNC) solution reached an approximately neutral pH.

### 2.4. Preparation of CCNC-Silica Hydrogel

A mixture comprising 2.8 mL of 1.7 wt% carboxylate cellulose nanocrystal (CCNC) dispersion (equivalent to 0.12 g dry cellulose), 9.2 mL of water, 2 mL of tetraethyl orthosilicate (TEOS) (98% purity, 1.9 g, 9.1 mmol), and 0.16 mL of aqueous HCl (0.29 M, 46 µmol HCl) was stirred overnight to facilitate the hydrolysis of TEOS, forming a CCNC-silica sol. Subsequently, 0.85 mL of 0.1 mol/L NH_3_ (85 µmol) was quickly added and thoroughly mixed to initiate condensation. The solution was then transferred into syringes to mould cylindrical hydrogels, with the flexibility to create different shapes using various moulds. After complete gelation, the silica nanocomposites were aged in water at 50 °C for a minimum of 10 h to increase the stiffness of the silica gel network. The hydrogels were then stored in distilled water in a refrigerator until further use.

### 2.5. Preparation of Titanium Dioxide

Titanium dioxide was synthesized using the sol–gel method as described by Marien et al. [16], known for its high yield of nanomaterials. A volume of 3 mL of titanium tetra-isopropoxide (precursor) was mixed with 20 mL of anhydrous ethanol to form Solution A. Solution B was prepared by mixing 13 mL of acetic acid with 2.5 mL of distilled water. Solution B was then added dropwise to Solution A until a transparent solution was obtained. After several hours, the sol turned milky, forming a gel, which was dried at 110 °C overnight to remove volatile solvents. The dried product was calcined at 500 °C to obtain titanium dioxide crystals.

### 2.6. Preparation of CCNC-Silica-TiO_2_ Hybrid Composite Aerogel

The CCNC-silica hydrogel prepared earlier (refer to Section 2.4) was immersed in a 2 wt% titanium dioxide (TiO_2_) sol–gel solution (refer to Section 2.5) at room temperature for 24 h with continuous stirring using a laboratory shaker. The mixture was then subjected to sonication at 70 °C for 4 h to achieve adsorption equilibrium. The resulting CCNC-silica- TiO_2_ hybrid composite hydrogel was thoroughly rinsed with distilled water and tertiary butyl alcohol (TBA) to remove any unreacted residues. Finally, the CCNC-silica-TiO_2_ hybrid composite aerogel was obtained by vacuum drying at 30 °C.

### 2.7. Characterization Methods

Fourier Transform Infrared Spectroscopy.

The samples were analyzed using Fourier transform infrared spectroscopy (FTIR) with a Perkin Elmer attenuated total reflection FTIR spectrometer (Perkin Elmer UATR Two, Johannesburg, South Africa) operating in diffuse reflectance mode. The spectral analysis covered a range from 4000 to 500 cm^−1^.

X-ray Diffraction.

The samples underwent X-ray diffraction (XRD) analysis using a Bruker AXS Advance D8 diffractometer located in Karlsruhe, Germany. The instrument utilized monochromatic Cu Kα radiation (λ = 1.5406 Å) as the X-ray source, operating at 40 kV and 40 mA under ambient temperature conditions. The crystallinity index (CI) was determined using both the Segal empirical method and the deconvolution method. The Segal empirical method calculates the CI based on the heights of *I*_002_ and *I*_min_, positioned between the 002 and 001 peaks. The CI calculation followed the procedure outlined below [17]:

(1) $$CI\;\%=\frac{I_{002}-I_{am}}{I_{002}}\times 100$$

Here, *I*_002_ stands for the maximum diffraction intensity of the peak, whereas *I*_am_ represents the intensity of diffraction from the amorphous material [17].

The deconvolution method determines the CI by analyzing the ratio between the combined area of all crystalline peaks and the total area.

(2) $$CI\;\%=\frac{{ƩA}_{cryst}}{{ƩA}_{cryst}+{ƩA}_{amorp}}\times 100$$

Here, A_cryst_ refers to the area corresponding to the crystalline domain, while A_amorp_ indicates the area associated with the amorphous domain in this context.

Scanning Electron Microscopy.

SEM imaging of the samples was conducted using an FEI Quanta 200 (Hillsboro, OR, USA) electron microscope operating at an acceleration voltage of 20 kV. Before imaging, the samples underwent carbon coating using Edward’s E306A coating system.

Thermogravimetric Analysis.

The samples underwent thermogravimetric analysis (TGA) using a Perkin Elmer Pyris 6 TGA analyser (Johannesburg, South Africa). Samples weighing between 10 and 15 mg were heated from 35 to 700 °C at a rate of 5 °C per minute. The analysis took place under a nitrogen atmosphere with a flow rate of 20 mL/min.

Brunauer–Emmett–Teller (BET).

Porosity and surface area were evaluated with a Micromeritics ASAP 2020 (Tristar II. Manufactured by Micromeritics, Norcross, GA, USA) surface area analyser. Prior to analysis, the samples were degassed at 100 degrees Celsius for 12 h under vacuum to remove moisture and gases.

### 2.8. Photocatalytic Activity

Batch photocatalytic degradation experiments of methylene blue (MB) were performed to evaluate the photodegradation process. A specific amount of photocatalyst (between 0.05 and 0.2 g) was added to 50 mL of MB dye solution, with initial concentrations ranging from 200 to 600 mg/L and exposed to UV light (a low-pressure mercury vapour lamp with a primary emission wavelength of 254 nm). The pH of the solution was adjusted from 2 to 12 using 0.1 M HCl or NaOH. The experiments were conducted at a constant temperature of 301 K with continuous stirring. At predetermined intervals (30, 60, 90, 120, 150, 180, 210, and 240 min), the remaining MB dye concentration was measured at 665 nm using a Pharo 300 Spectroquant UV-Vis spectrophotometer (Merck, Johannesburg, South Africa). Notably, the first 30 min occurred in darkness. For the parameter optimization studies (effects of pH, initial concentration, and catalyst dose), the total irradiation time was 150 min, unless otherwise stated. The degradation percentage (D%) and degradation concentration (qt) were calculated using the following equations:

(3) $$D\;(\%)=\frac{C_{0}-C_{t}}{C_{0}}\times 100$$

(4) $$qt\left(\frac{mg}{g}\right)=\left(C_{0}-C_{t}\right)\times \frac{V}{M}$$

where *C*_0_ and *C_t_* (mg/L) represent the MB concentration at the initial (0) time and time, respectively. V is the volume of the MB solution (L), and M is the net weight of the photocatalyst (g).

## 3. Results and Discussion

### 3.1. FTIR Spectroscopy Analysis

**Figure 1:**
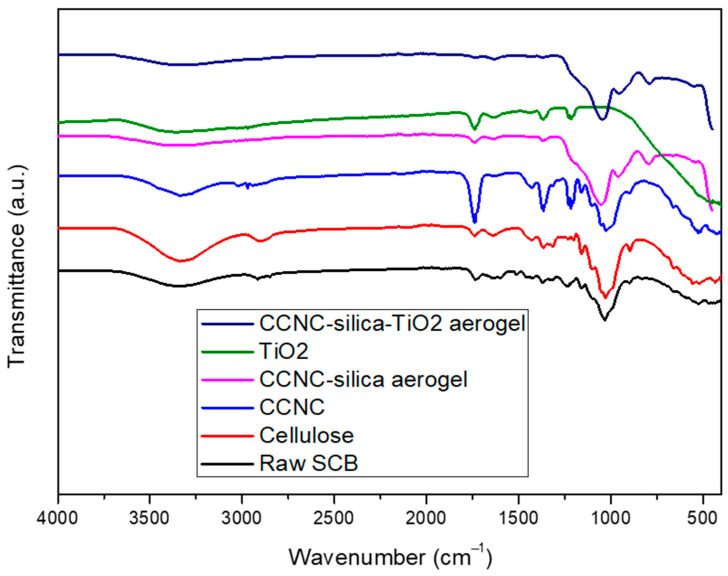
FTIR spectra of raw SCB, extracted cellulose, carboxylated cellulose, carboxylated CNC-silica aerogel, TiO_2,_ and carboxylated CNC-silica-TiO_2_ hybridized composite material.

FTIR analysis was performed to verify the synthesis of the carboxylate cellulose-based silica titanium dioxide (TiO_2_) hybrid nanocomposite. The FTIR spectra presented in Figure 1 includes data for raw sugarcane bagasse (SCB), extracted cellulose, carboxylate cellulose nanocrystals (CCNC), CCNC-silica aerogel, TiO_2_, and the CCNC-silica-TiO_2_ aerogel. In raw SCB, characteristic peaks indicative of natural fibres were observed at 3325 cm^−1^ (-OH stretching), 2896 cm^−1^ (C-H vibrations), 1361 cm^−1^ (-CH_2_ bending), 1025 cm^−1^ (C-O stretching), and 897 cm^−1^ (-CH_2_ bending), corresponding to cellulose I [18].

An additional peak at approximately 1312 cm^−1^ was distinctly present in extracted cellulose and CCNC but absent in raw SCB. This peak is likely due to C-H wagging, attributed to disrupted hydrogen bonds [19]. Extracted cellulose showed similar peak patterns to raw SCB, with the addition of the 1312 cm^−1^ peak. However, it lacked the peaks at 1508 and 1239 cm^−1^, while exhibiting increased intensity at 897 cm^−1^, indicating the removal of amorphous regions and the presence of β-glycosidic linkages between glucose units in cellulose [19,20].

In the FTIR spectrum of CCNC, a significant increase in the peak intensity of the carbonyl group at 1740 cm^−1^ was observed [21]. The FTIR spectra of the CCNC-silica aerogel displayed a distinct peak at 793 cm^−1^, attributed to the stretching and bending vibration of the silanol group (Si-OH) [22]. Additionally, peaks at 1361 cm^−1^, 1215 cm^−1^, and 1044 cm^−1^ were associated with C-O alkoxy stretching, C-O-C asymmetric stretch vibration, and C-O epoxy stretching, respectively, confirming the successful preparation of cellulose-based silica aerogels.

The presence of a broad O-H stretching peak (3200–3550 cm^−1^) indicated the presence of hydroxyl groups from both cellulose and possibly surface hydroxyl groups on TiO_2_. The decrease in intensity of the C=O stretching peak around 1720 cm^−1^, compared to the increase in intensity of the peak at 1640 cm^−1^, indicates the presence of O-H groups, likely from cellulose and surface hydroxyls on TiO_2_. The emergence of a new peak around 550 cm^−1^, attributed to Ti-OH stretching, confirms the presence of TiO_2_ within the composite.

### 3.2. X-Ray Diffraction Analysis

**Figure 2:**
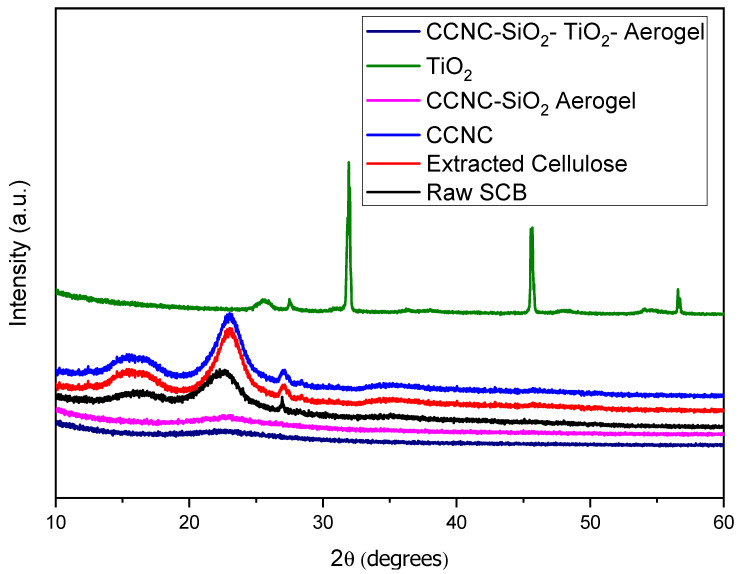
XRD spectra of raw SCB, extracted cellulose, carboxylated cellulose, carboxylated CNC-silica aerogel, TiO_2,_ and carboxylated CNC-silica-TiO_2_ aerogel hybridized composite material.

The X-ray diffraction analysis of raw sugarcane bagasse extracted cellulose, carboxylate cellulose nanocrystals (CCNC), CCNC-SiO_2_ aerogel, titanium dioxide (TiO_2_), and CCNC-SiO_2_-TiO_2_ aerogels reveal distinct structural characteristics at each stage of material processing and synthesis (Figure 2). The XRD pattern of raw sugarcane bagasse shows broad peaks around 2θ = 15° (110 plane) and 22° (200 plane), indicative of its semi-crystalline nature due to the presence of cellulose, hemicellulose, and lignin [23]. Upon extracting cellulose, the XRD pattern exhibits sharper peaks at 2θ = 22.5° (200 plane), reflecting the crystalline cellulose I structure and indicating the removal of amorphous hemicellulose and lignin [24]. For CCNC, a prominent peak at 2θ = 22.5° (200 plane) is observed, suggesting a higher degree of crystallinity due to the nanocrystals ordered structure [25]. The CCNC-SiO_2_ aerogel retains the characteristic cellulose I peak at 2θ = 22.5° (200 plane) and introduces new broad peaks around 2θ = 20° and 26°, corresponding to the amorphous silica network, indicating successful integration of SiO_2_ [26].

The XRD pattern of TiO_2_ shows distinct peaks at around 2θ = 25° (101 plane), 31° (004 plane), 46° (200 plane), and 56° (105 plane), representing the anatase phase of TiO_2_ [25,27]. The CCNC-SiO_2_-TiO_2_ aerogel combines features from both CCNC-SiO_2_ and TiO_2_, with the presence of the characteristic cellulose I peak at 2θ = 22.5° (200 planes), amorphous SiO_2_ peaks around 2θ = 20° and 26°, and the anatase TiO_2_ peaks at 2θ = 25°, 31°, 46°, and 56°, confirming the successful formation of a composite aerogel with enhanced properties [28].

These changes in the XRD patterns are quantified by the Crystallinity Index (CI), which increases from raw bagasse to extracted cellulose and decreases with each subsequent modification (Table 1). Carboxylate cellulose, formed by introducing carboxyl groups, shows a partial disruption of crystallinity with broader and less intense peaks, indicating partial amorphization [29]. The addition of amorphous silica and TiO_2_ further decreases the CI as observed in the aerogels. While peak positions remain relatively consistent, the intensities and widths of these peaks change, indicating the varying degrees of crystallinity and structural disruption through each processing stage. These results highlight the structural transformations critical for understanding the material properties and potential applications of modified cellulose derived from sugarcane bagasse [30].

### 3.3. SEM Analysis

**Figure 3:**
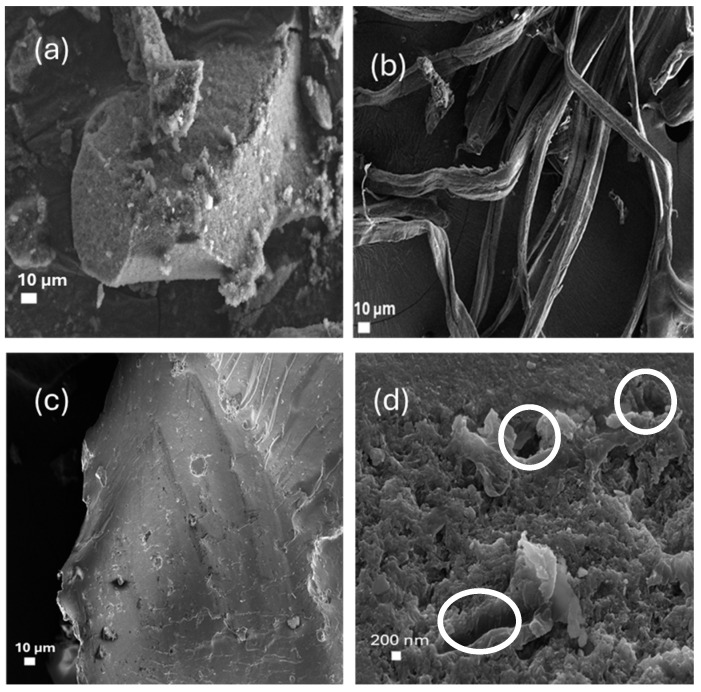
SEM images of (**a**) raw SCB, (**b**) extracted cellulose, (**c**,**d**) carboxylated CNC-silica-TiO_2_ aerogel hybridized composite material at different magnifications.

Figure 3 displays SEM micrographs depicting the morphological evolution of samples from raw sugarcane bagasse (SCB) to the synthesized CCNC-SiO_2_-TiO_2_ hybrid aerogel. The unrefined SCB (Figure 3a) has a dense and robust fibre structure, typical of lignocellulosic biomass, with noticeable surface impurities and flaws. Following cellulose extraction (Figure 3b), the structure exhibits a more refined and fibrillated appearance, indicating the effective removal of hemicellulose and lignin. In contrast, the CCNC-SiO_2_-TiO_2_ aerogel (Figure 3c,d) exhibits a highly porous, sponge-like architecture characterized by interlinked networks and open channels. The porous shape results from the incorporation of SiO_2_ and TiO_2_, which prevents the aggregation of CCNCs and create a robust 3D aerogel structure. The development of porous networks is crucial for improving photocatalytic activity, as they offer increased accessibility to active sites and promote the transport and adsorption of dye molecules on the aerogel surface [11,12].

### 3.4. TGA

**Figure 4:**
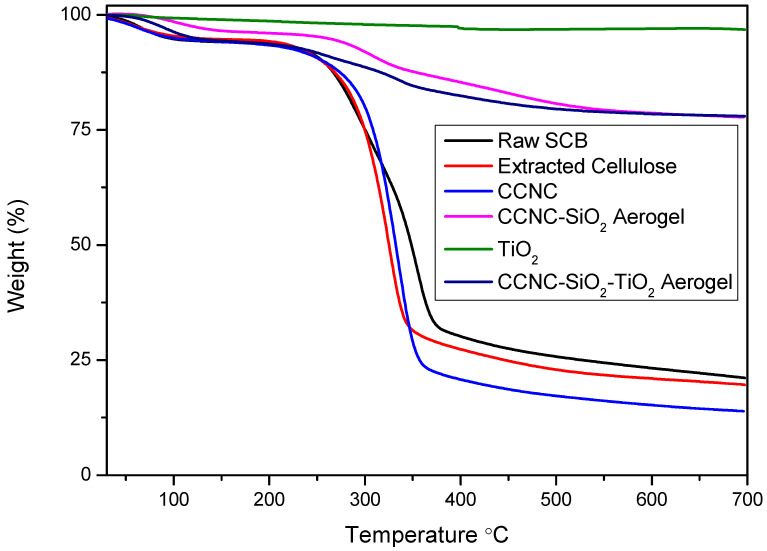
TGA spectra of raw SCB, extracted cellulose, carboxylated cellulose, carboxylated CNC-silica aerogel, TiO_2,_ and carboxylated CNC-silica-TiO_2_ aerogel hybridized composite material.

**Figure 5:**
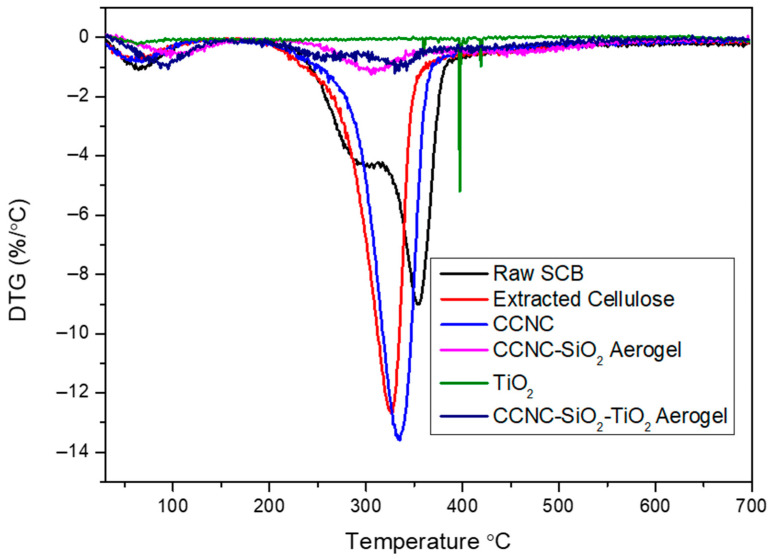
DTG spectra of raw SCB, extracted cellulose, carboxylated cellulose, carboxylated CNC-silica aerogel, TiO_2,_ and carboxylated CNC-silica-TiO_2_ aerogel hybridized composite material.

The TGA and DTG analysis provide a comprehensive understanding of the thermal stability, decomposition behaviour, and residual or ash content of raw sugarcane bagasse, extracted cellulose, carboxylate cellulose nanocrystals (CCNC), CCNC-SiO_2_ aerogel, titanium dioxide (TiO_2_), and CCNC-SiO_2_-TiO_2_ aerogels (Figure 4 and Figure 5). The TGA plot for raw sugarcane bagasse typically exhibits a three-step degradation process. The initial weight loss of around 100 °C corresponds to the evaporation of moisture. The second significant weight loss occurring between 270 and 390 °C is associated with the degradation of hemicellulose and part of the cellulose. The third weight loss observed around 320–354 °C is due to the degradation of cellulose and lignin. The DTG plot typically shows two pronounced peaks: one around 300 °C representing hemicellulose degradation and another around 354 °C corresponding to cellulose degradation [31,32]. The significant residual or ash content left after complete thermal degradation indicates the presence of inorganic components.

Extracted cellulose demonstrates improved thermal stability compared to raw sugarcane bagasse. The TGA plot shows minimal initial weight loss, indicating low moisture content. The primary decomposition occurs between 300 and 326 °C due to cellulose degradation, with the DTG plot displaying a single prominent peak around 320–325 °C [33,34]. The residual content is lower than that of raw bagasse, reflecting the removal of inorganic impurities during the extraction process. CCNC exhibits higher thermal stability compared to extracted cellulose. The TGA plot shows a primary weight loss between 310 and 334 °C, attributed to the decomposition of carboxylate groups and cellulose. The DTG plot features a main peak around 335–340 °C [12,35]. The relatively low residual content indicates the effective removal of non-cellulosic components during the production of nanocrystals.

The incorporation of SiO_2_ into CCNC further enhances thermal stability. The TGA plot shows minimal initial weight loss due to moisture. The primary weight loss occurs between 400 and 453 °C, associated with the decomposition of cellulose and the organic matrix. The DTG curve typically displays a broad peak around 300–370 °C, indicating the decomposition of organic components while the inorganic SiO_2_ remains stable [36]. The high residual content is due to the presence of thermally stable SiO_2_. TiO_2_ demonstrates excellent thermal stability, with minimal weight loss observed in the TGA plot up to very high temperatures. The DTG plot generally shows no significant peaks, indicating that TiO_2_ does not decompose within the tested temperature range [37]. The residual content is nearly 100%, reflecting the high thermal stability and inert nature of TiO_2_.

The TGA plot of CCNC-SiO_2_-TiO_2_ aerogels indicates enhanced thermal stability compared to the individual components. The initial weight loss is minimal, suggesting low moisture content. The major weight loss occurs between 300 and 400 °C, corresponding to the decomposition of cellulose, the organic matrix, and any residual carboxylate groups. The DTG curve typically shows a broad peak around 320–380 °C, reflecting the combined thermal degradation of cellulose, organic matrix, and some interactions between SiO_2_ and TiO_2_ [38,39]. The residual content of the CCNC-SiO_2_-TiO_2_ aerogel is significant and comparable to that of the CCNC-SiO_2_ aerogel. This observation can be explained by considering the synthesis pathway. The TiO_2_ was incorporated by immersing the pre-formed CCNC-SiO_2_ hydrogel into a TiO_2_ sol–gel solution. During this step, some Ti-O-Si bonds likely formed at the interface, integrating the TiO_2_ into the existing silica network [38,39]. Furthermore, the final composite was vacuum-dried but not recalcined after the TiO_2_ incorporation step. Therefore, the titania component likely exists as an amorphous or poorly crystalline titania-silica mixed oxide, rather than as pure, dense anatase TiO_2_. The residual mass in TGA represents the final, thermally stable inorganic ash. Since the silica framework forms the primary inorganic scaffold in both aerogels, and the incorporated titanium species are integrated into this matrix without adding a large mass of pre-formed, crystalline TiO_2_, the total inorganic residue after organic combustion appears similar. The key difference lies not in the final ash quantity but in the composition of that ash, which now includes titanium species alongside silica, contributing to enhanced photocatalytic activity.

The TGA and DTG analyses reveal that the thermal stability and decomposition behaviour of these materials vary significantly based on their composition. Raw sugarcane bagasse shows multiple degradation stages with high residual content, while extracted cellulose and CCNC demonstrate improved thermal stability with lower residual content. The incorporation of SiO_2_ and TiO_2_ into CCNC enhances thermal stability and increases the residual content due to the presence of thermally stable inorganic components, making these composites suitable for high-temperature applications.

### 3.5. BET Analysis

**Figure 6:**
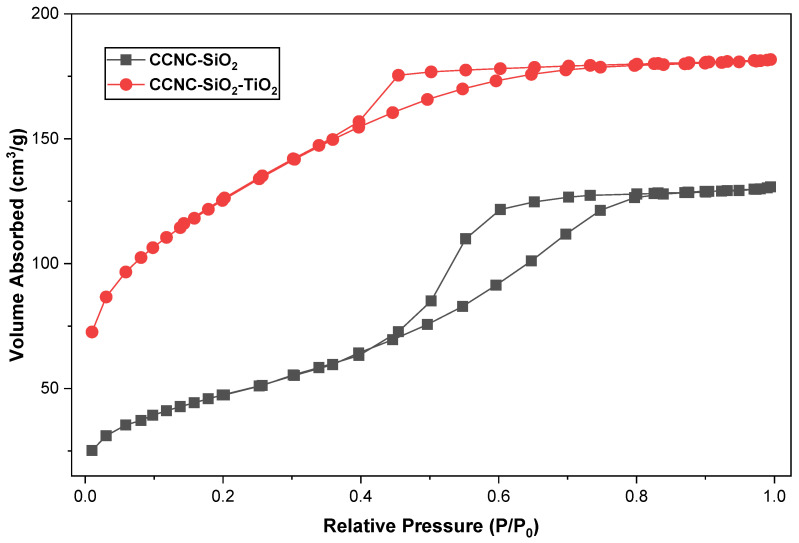
Nitrogen adsorption and desorption isotherms plots of CCNC-SiO_2_ and CCNC-SiO_2_-TiO_2_ aerogels.

The BET study, illustrated in Figure 6 and Table 2, substantiates the porosity and surface characteristics observed in the SEM images. The nitrogen adsorption–desorption isotherms for CCNC-SiO_2_ and CCNC-SiO_2_-TiO_2_ aerogels are classified as type IV, exhibiting H3 hysteresis loops, which are characteristic of mesoporous materials [13]. The CCNC-SiO_2_ aerogel possesses a specific surface area of 312 m^2^/g, a pore volume of 0.12 cm^3^/g, and an average pore diameter of 1.15 nm, thereby affirming its mesoporous properties. The integration of TiO_2_ yields values of 448.69 m^2^/g, 0.30 cm^3^/g, and 2.49 nm, demonstrating that TiO_2_ nanoparticles effectively merge with the silica-cellulose matrix, hence enhancing pore channels and texture. The increased surface area and pore volume improve molecular adhesion, whereas the larger pore diameter promotes the mobility of dye molecules in photocatalysis [14,38]. The SEM and BET analyses demonstrate that the hybrid aerogel features a unique mesoporous structure and a substantial surface area, both essential for its enhanced photocatalytic degradation efficacy.

### 3.6. Factors Affecting Methylene Blue Dye Adsorption

#### 3.6.1. Effect of Contact Time

**Figure 7:**
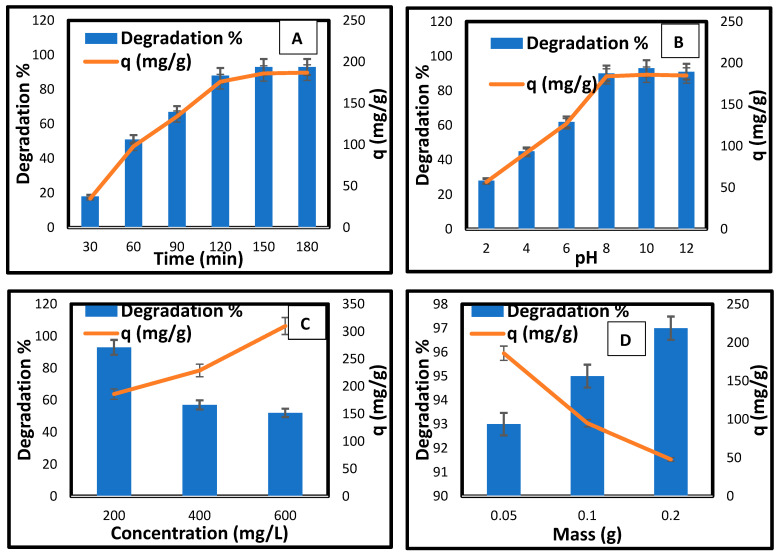
(**A**) Effect of contact time, (**B**) pH, (**C**) initial concentration, and (**D**) photocatalyst dose on the photodegradation of MB by CCNC-silica-TiO_2_ aerogel.

In Figure 7A, the effect of contact time on the photocatalytic degradation of methylene blue using CCNC-silica-TiO_2_ aerogel was investigated to determine the optimal irradiation duration for subsequent experiments. The degradation efficiency showed a marked improvement with increased exposure time, but with a notable distinction from the initial 30 min spent in the dark. During this initial dark phase, the removal percentage is 18%, with an adsorption capacity of 35 mg/g. This indicates significant adsorption of MB onto the aerogel’s surface and pores, establishing adsorption equilibrium prior to light irradiation. Once the light exposure begins, the degradation rate accelerates, with degradation percentages rising to 51% at 60 min (98 mg/g), 67% at 90 min (134 mg/g), and 88% at 120 min (176 mg/g). The maximum degradation percentage of 93% and a degradation capacity of 186 mg/g are achieved at both 150 and 180 min, showing that the photocatalytic process continues effectively without further improvement after 150 min (Table 3). This suggests that while the initial dark period limits photocatalytic activity, once light is introduced, the CCNC-silica-TiO_2_ aerogel significantly enhances the degradation of methylene blue, reaching optimal performance by 150 min.

#### 3.6.2. Effect of pH

The effect of pH on the photocatalytic degradation of methylene blue using CCNC-silica-TiO_2_ aerogel (Figure 7B) was evaluated after 150 min of irradiation, the time determined to be optimal for maximum degradation. The results reveal a strong influence of pH on the efficiency of the degradation process. At pH 2, the degradation percentage of methylene blue is relatively low at 28%, corresponding to a degradation concentration of 57 mg/g, indicating that acidic conditions hinder photocatalytic activity. As the pH increases to 4, the degradation percentage improves to 45% with a degradation concentration of 92 mg/g, showing that the degradation efficiency starts to rise. This trend continues up to pH 6, where the removal percentage reaches 62% and the quantity of methylene blue degraded is 127 mg/g. The photocatalytic activity significantly enhances at neutral and slightly alkaline conditions, peaking at pH 8 with a removal percentage of 90% and a degradation concentration of 184 mg/g. At pH 10, the removal percentage reaches its highest at 93%, with a degradation concentration of 186 mg/g, and remains nearly constant at pH 12 with a removal percentage of 91% and 185 mg/g. These results suggest that the CCNC-silica-TiO_2_ aerogel performs optimally under slightly alkaline to neutral conditions, with pH 10 providing the most efficient degradation of methylene blue, while more acidic conditions adversely affect the photocatalytic activity.

#### 3.6.3. Effect of Initial Methylene Blue Concentration

Figure 7C illustrates the effect of initial methylene blue (MB) concentration on the photocatalytic degradation using CCNC-silica-TiO_2_ aerogel, demonstrating a clear inverse relationship between dye concentration and degradation efficiency. At an initial concentration of 200 mg/L, the removal percentage is high, at 93%, with a degradation concentration of 186 mg/g of methylene blue, indicating effective photocatalytic activity. However, as the concentration increases to 400 mg/L, the removal percentage drops to 57%, even though the degradation concentration of the dye removed increases to 229 mg/g. This trend continues at an initial concentration of 600 mg/L, where the degradation percentage further decreases to 52%, but the degradation concentration of methylene blue rises to 310 mg/g. These results suggest that higher initial concentrations of methylene blue lead to a lower degradation efficiency, likely due to the saturation of active sites on the photocatalyst and increased competition among dye molecules for the reactive sites [40]. Despite the lower degradation efficiency at higher concentrations, the total amount of dye removed increases, indicating that the photocatalyst is still effective in processing larger amounts of dye but becomes less efficient at higher concentrations [41].

#### 3.6.4. Effect of Photocatalyst Dose

The effect of photocatalyst dose on the photocatalytic degradation of methylene blue using CCNC-silica-TiO_2_ aerogel (Figure 7D) exhibits an interesting trend. Initially, increasing the adsorbent dose enhance the degradation efficiency, but eventually, it leads to a decrease in the degradation concentration of dye removed per gram of adsorbent. At an adsorbent dose of 0.05 g, the degradation percentage of methylene blue is high at 93%, with a degradation concentration of 186 mg/g of dye removed. Increasing the dose to 0.1 g improves the degradation efficiency slightly to 95%, but the degradation concentration of the dye removed decreases to 95 mg/g. Further increasing the dose to 0.2 g results in a peak degradation percentage of 97%, yet the degradation concentration of dye removed per gram of adsorbent drops significantly to 48 mg/g. This pattern suggests that while a higher adsorbent dose increases the overall degradation efficiency, it also leads to a lower specific degradation concentration, likely due to a higher number of available active sites that dilute the concentration of methylene blue, reducing the per-gram efficiency [42]. The optimal adsorbent dose appears to be around 0.1 g, where the balance between degradation efficiency and specific capacity is most favourable.

### 3.7. Photocatalytic/Adsorption Kinetics

The kinetics of the photocatalytic degradation process offer valuable insights into the mechanism underlying the removal of MB dye using the carboxylate cellulose nanocrystal-silica-titanium dioxide (CCNC-silica-TiO_2_) hybrid aerogel. To analyze the photodegradation behaviour, the pseudo-first-order kinetic model was applied, based on the Langmuir–Hinshelwood mechanism at low contaminant concentrations. This model assumes that the degradation rate is directly proportional to the concentration of the contaminant (in this case, MB dye). The process is described by the following equation:

(5) $$ln\left(\frac{C_{0}}{C_{t}}\right)=kt$$

Here, *C*_0_ represents the initial concentration of MB, *C_t_* is the concentration at time *t*, and *k* denotes the first-order rate constant. This behaviour is characteristic of heterogeneous photocatalytic systems such as CCNC-silica-TiO_2_, where adsorption and photocatalytic degradation occur simultaneously. In the CCNC-silica-TiO_2_ hybrid aerogel, the high surface area of carboxylate cellulose nanocrystals (CCNCs) and silica enhances the adsorption of MB dye, while TiO_2_ drives photocatalytic activity under UV light. The synergistic effect of adsorption and photocatalysis significantly improves the MB dye degradation efficiency.

**Figure 8:**
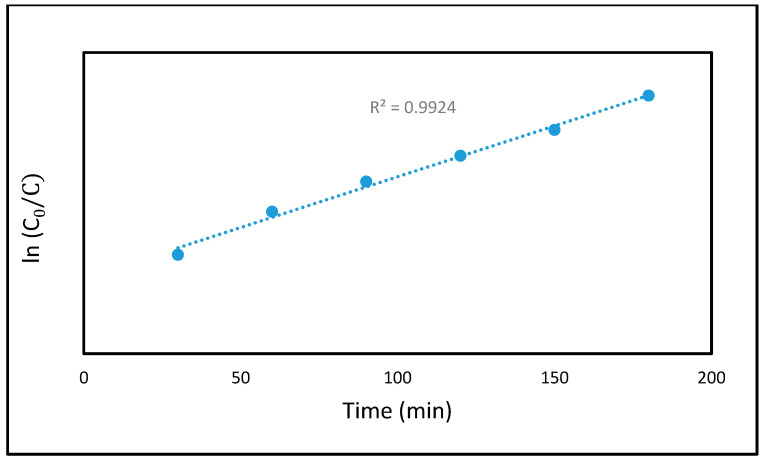
Pseudo-first order kinetics plot of MB dye degradation on CCNC-silica-TiO_2_ aerogel.

The Langmuir–Hinshelwood kinetic model, widely used in heterogeneous photocatalysis, effectively describes the interaction between MB adsorption on the CCNC-silica-TiO_2_ aerogel surface and their subsequent photocatalytic degradation. As shown in Figure 8, the reaction kinetics of MB dye degradation in the presence of the CCNC-silica-TiO_2_ aerogel were analyzed by plotting *ln* (*C*_0_/*C*) vs. *t*. The resulting straight line confirms that the photocatalytic degradation of MB dye follows first-order kinetics, with a correlation coefficient (R^2^) exceeding 0.95. A strong correlation to first-order kinetics was observed for the CCNC-Silica-TiO_2_ aerogel (R^2^ = 0.993), with a rate constant of kapp = 0.0144 min^−1^. This value is significantly higher than those measured for the CCNC-Silica aerogel and pure TiO_2_ nanoparticles (0.0015 and 0.0080, respectively), quantitatively demonstrating the superior photocatalytic activity of the hybrid composite due to the synergistic effect between its components. These findings demonstrate that the CCNC-silica-TiO_2_ photocatalyst exhibits high photo-reactivity, further supporting its effectiveness in MB dye degradation.

#### Photocatalyst Recycling Test

**Figure 9:**
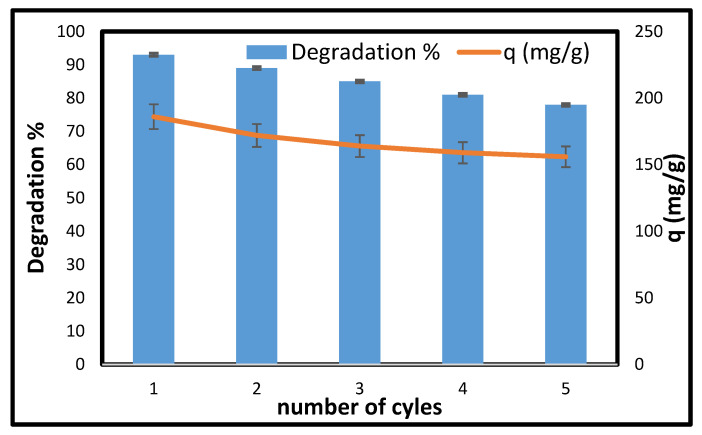
Reusability of CCNC-silica-TiO_2_ for the photodegradation of MB in water. [Conc. 200 mg/L, Volume 50 mL, pH 10, Mass 0.05 g, and Temperature 25 °C].

The economic importance of a stable and easily regenerable adsorbent is crucial, making reusability a vital consideration. In light of this, the CCNC-silica-TiO_2_ aerogel was tested across multiple photocatalysis cycles. As illustrated in Figure 9, the degradation capacity remained above 70%, with a peak degradation concentration of 148 mg/g after five consecutive cycles. This demonstrates the excellent stability and remarkable reusability of the synthesized CCNC-silica-TiO_2_ aerogel.

### 3.8. Proposed Photocatalytic Degradation Performance Comparison with Other Adsorbents

The exceptional photocatalytic activity of the CCNC-SiO_2_-TiO_2_ aerogel results from a synergistic mechanism that enhances adsorption and improves photocatalysis. The procedure commences with the aerogel’s highly porous, three-dimensional architecture, as validated by SEM and BET measurements. This structure enables MB dye molecules to rapidly disperse and adhere to its extensive surface area. The carboxylate groups (-COO^−^) on the CCNC surface are crucial for this process as they offer robust electrostatic interaction sites for the cationic MB dye, thereby localizing the pollutant to the active photocatalytic sites [43].

When ultraviolet light interacts with TiO_2_, photons possessing energy equivalent to or above the bandgap (about 3.2 eV for anatase) promote electrons (e^−^) from the valence band (VB) to the conduction band (CB), resulting in the formation of electron-hole (h^+^) pairs. The silica matrix and the integrated CCNC network prevent the recombination of photogenerated charge carriers by providing alternative pathways for their movement and locations for their entrapment [13,38].

The subsequent processes involving adsorbed water and oxygen molecules generate highly reactive oxygen species (ROS). The holes generated by light (h^+^) can directly oxidize MB dye or react with H_2_O or OH^−^ on the surface to produce hydroxyl radicals (•OH). Simultaneously, conduction band electrons (e^−^) can reduce adsorbed molecular oxygen (O_2_) to form superoxide anion radicals (•O_2_^−^). These radicals can subsequently undergo protonation to become hydroperoxyl radicals (OOH), which can further generate more •OH radicals [9,10,44].

The degradation of MB is mostly attributed to the assault of highly oxidizing species (•OH and h^+^, and to a lesser degree •O_2_^−^) on it. The proposed degradation process involves a series of complex events, leading to the rupture of the conjugated chromophore structure, hence causing the disappearance of the blue colour. Subsequently, N-demethylation processes occur, systematically eliminating methyl groups from the dimethylamino functional groups of MB. Azure A, Azure B, Azure C, and Thionine are identified as intermediate products [45]. Increased oxidative assaults disrupt the aromatic rings, converting the molecule into diminutive, colourless organic acids, ultimately mineralizing it into innocuous end products such as CO_2_, H_2_O, SO_4_^2−^, and NO_3_^−^ [46].

The integration of CCNC’s adsorption capacity and TiO_2_’s photocatalytic function, both stabilized within the silica aerogel matrix, creates a highly efficient system. The CCNC operates as a molecular antenna, collecting dye contaminants and transporting them to the TiO_2_ sites for mineralization. This significantly accelerates the overall degradation rate and efficiency.

### 3.9. Performance Comparison with Other Photocatalyst/Adsorbents

To further contextualize the performance of the CCNC-SiO_2_-TiO_2_ aerogel, its maximum MB adsorption capacity (186 mg/g) was quantitatively compared with other recently developed bio-based and TiO_2_ composite photocatalysts, as summarized in Table 4. While direct comparisons must be made cautiously due to differences in experimental conditions (e.g., initial concentration, catalyst dose, light source), the synthesized aerogel in this work demonstrates a highly competitive and superior degradation capacity. It outperforms numerous other composites composed of cellulose or carbon and performs comparably to specific contemporary graphene-based materials. The aerogel’s distinctive synergistic structure imparts this additional capability. The silica matrix provides a substantial surface area and mesoporosity, the carboxylated CNC enhances dye preconcentration efficiency, and the uniformly dispersed, accessible TiO_2_ nanoparticles facilitate photocatalysis. The proposed CCNC-SiO_2_-TiO_2_ hybrid system is novel and possesses significant promise for effective wastewater treatment due to its high adsorption capacity derived from sustainable sugarcane bagasse and exceptional photocatalytic activity.

## 4. Conclusions

This study successfully developed and studied a novel CCNC-SiO_2_-TiO_2_ hybrid composite aerogel for the efficient photocatalytic degradation of methylene blue. The synthesis method transformed waste sugarcane bagasse into functionalized cellulose nanocrystals, which were later integrated into a porous silica matrix with TiO_2_ nanoparticles. A thorough analysis indicated that the material exhibited the suitable chemical composition, enhanced thermal stability, and ideal physical properties, such as a large surface area and a mesoporous structure favourable for pollutant interaction. The composite aerogel demonstrated exceptional photocatalytic efficacy, degrading 93% of MB under optimal conditions. This far surpasses the capabilities of its non-photocatalytic predecessor. The process was determined to adhere to pseudo-first-order kinetics. The aerogel demonstrated remarkable stability and the ability to be reused. The improved performance results from a synergistic mechanism: the carboxylate CNC surface proficiently adsorbs MB dye molecules and guides them to the TiO_2_ sites, where they are afterward mineralized by UV radiation. This research demonstrates the application of agricultural waste in producing innovative, multifunctional materials. The CCNC-SiO_2_-TiO_2_ aerogel serves as an effective, durable, and efficient approach for the remediation of dye-contaminated wastewater. It demonstrates high efficiency and notable reusability.

This study demonstrates that the CCNC-SiO_2_-TiO_2_ aerogel can be synthesized in a laboratory setting and exhibits commendable performance; however, it is crucial to discuss its scalability for industrial applications. Large-scale production appears feasible; nonetheless, numerous issues require resolution. The primary materials available for purchase include sugarcane bagasse (an agricultural byproduct), silica precursors (such as TEOS), and titanium isopropoxide. The sol–gel and vacuum drying techniques employed in materials research are widely recognized. The processes of centrifugation and dialysis for CNC purification, the utilization of expensive chemicals such as TEOS, and the extended durations of vacuum drying that consume significant energy will substantially impact the overall cost and energy footprint at scale. Future endeavours will concentrate on enhancing the synthesis for industrial applications, including the exploration of more economical silica sources, the investigation of continuous flow processes for CNC production, and the implementation of expedited, energy-efficient drying methods such as supercritical CO_2_ drying to fabricate the aerogel structure. Despite these challenges, the utilization of an abundant, cost-free waste feedstock (bagasse), the aerogel’s exceptional reusability (exceeding five cycles), and its superior performance may offset production costs, rendering it a viable and sustainable solution for treating concentrated dye wastewater streams. The current reliance on UV light is a limitation of the pristine anatase TiO_2_ used. Future research will be directed toward modifying the CCNC-SiO_2_-TiO_2_ aerogel to enhance its visible-light responsiveness. Promising strategies include doping with non-metal elements (e.g., nitrogen, sulphur) or metals (e.g., iron, copper), and coupling with narrow-bandgap semiconductors (e.g., C_3_N_4_) or quantum dots to create heterojunctions that can be activated by visible light. Such modifications would be a significant step toward developing a truly solar-driven photocatalytic system for wastewater remediation.

## Figures and Tables

**Table 1:** Crystalline Index values of materials studied using Segal and deconvolution methods.

Fibre Type	CI (%) (Segal)	CI (%) (Deconvolution)
Raw SCB	29	27
Extracted cellulose	66	63
CCNC	58	54
CCNC-silica aerogel	-	42
CCNC-silica-TiO_2_ aerogel	-	23

**Table 2:** Physical parameters of the prepared aerogels.

Sample	Specific Surface Area (m^2^/g)	Pore Volume (cm^3^/g)	Pore Diameter (nm)
CCNC-SiO_2_	312	0.12	1.15
CCNC-SiO_2_-TiO_2_	448.69	0.30	2.49

**Table 3:** Photocatalytic degradation study results for cellulose-based silica aerogels studied.

Cellulose-Based Silica Aerogels	Removal (%)	Adsorption Capacity (mg/g)
CCNC silica	22	32
CCNC-silica-TiO_2_	93	186

**Table 4:** Quantitative comparison of the maximum MB adsorption/degradation capacity of the CCNC-SiO_2_-TiO_2_ aerogel with other reported bio-based and composite photocatalysts.

Photocatalyst Material	Maximum Capacity (mg/g)	Initial Conc. (mg/L)	Catalyst Dose (g/L)	Light Source	Reference
CCNC-SiO_2_-TiO_2_ (This work)	186	200	1.0	UV (254 nm)	-
Regenerated Cellulose/GO Aerogel	189	100	0.2	Adsorption	[42]
Chitosan/GO/Lignosulfonate Aerogel	205	200	0.4	Adsorption	[40]
Sodium Carboxymethyl Cellulose-based Adsorbent	176	200	1.0	Adsorption	[41]
g-C_3_N_4_/N-doped Cellulose Nanofiber Aerogel	123	50	0.5	Visible	[46]

## Data Availability

The original contributions presented in this study are included in the article. Further inquiries can be directed to the corresponding author.
